# Supplementary material for: Species turnover in plants does not predict turnover in flower-visiting insects
Source: PeerJ. 2018 Dec 21;6:e6139. doi: 10.7717/peerj.6139 (PMC6305123; doi:10.7717/peerj.6139)
Supplement: Supplemental Information 1 [file peerj-06-6139-s001.docx]

**Appendix S1** Distance table for all plots used in species turnover study.

| CO2 | 0.705 |  |  |  |  |  |  |  |  |  |  |  |  |  |  |
| --- | --- | --- | --- | --- | --- | --- | --- | --- | --- | --- | --- | --- | --- | --- | --- |
| EL1 | 67.135 | 66.740 |  |  |  |  |  |  |  |  |  |  |  |  |  |
| EL2 | 67.646 | 67.251 | 0.514 |  |  |  |  |  |  |  |  |  |  |  |  |
| EL3 | 67.635 | 67.225 | 1.874 | 1.762 |  |  |  |  |  |  |  |  |  |  |  |
| EL4 | 66.304 | 65.917 | 1.188 | 1.617 | 2.962 |  |  |  |  |  |  |  |  |  |  |
| HE1 | 3.866 | 3.652 | 69.317 | 69.825 | 69.732 | 68.526 |  |  |  |  |  |  |  |  |  |
| HE2 | 3.971 | 3.821 | 69.672 | 70.181 | 70.091 | 68.880 | 0.385 |  |  |  |  |  |  |  |  |
| HE3 | 3.171 | 3.089 | 69.323 | 69.832 | 69.762 | 68.520 | 0.911 | 0.842 |  |  |  |  |  |  |  |
| KL1 | 29.598 | 28.959 | 68.692 | 69.134 | 68.378 | 68.302 | 26.882 | 27.105 | 27.772 |  |  |  |  |  |  |
| GB1 | 37.692 | 37.095 | 75.516 | 75.934 | 75.046 | 75.219 | 34.621 | 34.792 | 35.53 | 9.083 |  |  |  |  |  |
| GB2 | 37.423 | 36.828 | 75.628 | 76.047 | 75.167 | 75.326 | 34.335 | 34.504 | 35.244 | 8.949 | 0.378 |  |  |  |  |
| GB3 | 37.455 | 36.867 | 76.278 | 76.699 | 75.825 | 75.972 | 34.321 | 34.482 | 35.231 | 9.303 | 1.017 | 0.720 |  |  |  |
| VG1 | 8.166 | 8.034 | 72.900 | 73.405 | 73.248 | 72.141 | 4.399 | 4.215 | 4.995 | 25.415 | 32.441 | 32.129 | 32.033 |  |  |
| VG2 | 7.388 | 7.251 | 72.271 | 72.777 | 72.631 | 71.507 | 3.617 | 3.433 | 4.218 | 25.652 | 32.82 | 32.512 | 32.430 | 0.783 |  |
| VG3 | 8.341 | 7.957 | 70.508 | 71.011 | 70.805 | 69.777 | 4.640 | 4.719 | 5.531 | 22.785 | 30.204 | 29.908 | 29.863 | 3.133 | 3.038 |
|  | CO1 | CO2 | EL1 | EL2 | EL3 | EL4 | HE1 | HE2 | HE3 | KL1 | GB1 | GB2 | GB3 | VG1 | VG2 |
| *Distances calculated in kilometres from GPS coordinates (WGS 84) of central pan trap array in each plot. | | | | | | | | | | | | | | | |
