# Supplementary material for: Species turnover in plants does not predict turnover in flower-visiting insects
Source: PeerJ. 2018 Dec 21;6:e6139. doi: 10.7717/peerj.6139 (PMC6305123; doi:10.7717/peerj.6139)
Supplement: Supplemental Information 2 [file peerj-06-6139-s002.docx]

**Appendix S2.** Insect and plant species identified from the study.

**Insect List.**

List of flower-visiting insects recorded in pan-trap surveys of six sites in the lowlands of the Cape Floristic region. Further details of the sites can be found in the main text. Arrangement of families follows that of Scholtz & Holm (2008).

|  | **Species/morphospecies** | **Abundances** | | | | | | |
| --- | --- | --- | --- | --- | --- | --- | --- | --- |
|  |  | **CO** | **EL** | **HE** | **KL1** | **PH** | **VG** | **Total** |
| **HEMIPTERA** |  |  |  |  |  |  |  |  |
| **Reduviidae** |  |  |  |  |  |  |  |  |
|  | Reduviid | 0 | 2 | 0 | 0 | 0 | 0 | 2 |
|  | *Rhinocoris* sp. | 0 | 0 | 0 | 0 | 1 | 0 | 1 |
| **Coreidae** |  |  |  |  |  |  |  |  |
|  | *Acanthoria* cf. *muricata* | 0 | 0 | 1 | 0 | 0 | 0 | 1 |
| **Lygaeidae** |  |  |  |  |  |  |  |  |
|  | *Dieuches* sp. | 13 | 0 | 5 | 0 | 6 | 1 | 25 |
|  |  |  |  |  |  |  |  |  |
| **HETEROPTERA** |  |  |  |  |  |  |  |  |
| **Pentatomidae** |  |  |  |  |  |  |  |  |
|  | Pentatomid | 0 | 0 | 0 | 0 | 0 | 1 | 1 |
|  |  |  |  |  |  |  |  |  |
| **COLEOPTERA** |  |  |  |  |  |  |  |  |
|  | Indet. Beetle sp.I | 0 | 0 | 1 | 0 | 0 | 0 | 1 |
| **Carabidae** |  |  |  |  |  |  |  |  |
|  | *Abacetus minutus* | 0 | 0 | 0 | 0 | 0 | 1 | 1 |
|  | *Abacetus perturbator* | 0 | 0 | 1 | 0 | 0 | 0 | 1 |
| **Staphylinidae** |  |  |  |  |  |  |  |  |
|  | Staphylinid sp.I | 1 | 8 | 0 | 0 | 0 | 3 | 12 |
| **Scarabaeidae** |  |  |  |  |  |  |  |  |
|  | *Anisochelus inornatus* | 0 | 87 | 0 | 0 | 0 | 0 | 87 |
|  | *Anisonyx* sp.I | 0 | 1 | 0 | 0 | 0 | 0 | 1 |
|  | *Anisonyx ursus* | 0 | 377 | 0 | 14 | 14 | 0 | 405 |
|  | *Campulipis limbatus* |  |  |  |  |  |  |  |
|  | *Dichelus* sp.I | 0 | 0 | 2 | 0 | 0 | 0 | 2 |
|  | *Pachynema* sp. | 0 | 0 | 0 | 0 | 5 | 5 | 10 |
|  | *Diaplochelus* sp. | 4 | 0 | 1 | 0 | 16 | 1 | 22 |
|  | *Dichelus (Heterochelus) arthriticus* | 2 | 0 | 8152 | 0 | 1106 | 1189 | 10449 |
|  | *Dichelus (Heterochelus) ditus* | 74 | 18 | 1 | 0 | 0 | 0 | 93 |
|  | *Dichelus (Heterochelus) forcipatus* | 0 | 0 | 0 | 41 | 41 | 0 | 82 |
|  | *Dichelus (Heterochelus) hybridus* | 0 | 0 | 0 | 0 | 3 | 0 | 3 |
|  | *Dichelus (Heterochelus) insignus* | 0 | 0 | 0 | 0 | 0 | 1 | 1 |
|  | *Dichelus (Heterochelus) rufimanus* | 0 | 0 | 8 | 0 | 121 | 92 | 221 |
|  | *Dichelus (Ischnochelus) sulcatus* | 0 | 6 | 0 | 0 | 0 | 0 | 6 |
|  | *Dichelus* cf. *acanthropus* | 99 | 111 | 3 | 0 | 0 | 0 | 213 |
|  | *Dichelus* cf. *denticeps* | 6 | 296 | 8 | 0 | 19 | 0 | 329 |
|  | *Dichelus* cf. *detritus* | 0 | 0 | 0 | 0 | 2 | 0 | 2 |
|  | *Dichelus* sp.I | 11 | 90 | 26 | 56 | 56 | 6 | 245 |
|  | *Dichelus* sp.II | 319 | 108 | 23 | 109 | 109 | 0 | 668 |
|  | *Kubousa axillaris* | 2 | 3 | 0 | 0 | 0 | 10 | 15 |
|  | *Kubousa gentilis* | 0 | 3 | 0 | 0 | 0 | 1 | 4 |
|  | *Lepithrix modesta* | 0 | 3 | 0 | 0 | 0 | 0 | 3 |
|  | *Lepithrix* sp.I | 0 | 0 | 2 | 0 | 0 | 0 | 2 |
|  | *Macrodicranocnemus andreaei* | 0 | 0 | 0 | 12 | 12 | 0 | 24 |
|  | *Monochelus elandsbergensis* | 4 | 1 | 0 | 0 | 0 | 0 | 5 |
|  | *Omocrates* sp. | 5 | 4 | 0 | 10 | 10 | 275 | 304 |
|  | *Pachynema crassipes* | 0 | 298 | 0 | 0 | 0 | 0 | 298 |
|  | *Pachynema saga* | 0 | 0 | 0 | 0 | 62 | 0 | 62 |
|  | *Peritrichia abdominalis* | 1 | 128 | 11 | 51 | 51 | 2 | 244 |
|  | *Peritrichia* cf. *pistinaria* | 13 | 0 | 97 | 0 | 8 | 77 | 195 |
|  | *Platychelus* cf. *caffer* | 1 | 15 | 1 | 0 | 0 | 0 | 17 |
|  | *Platychelus* cf. *karrooensis* | 0 | 37 | 0 | 0 | 0 | 1 | 38 |
|  | *Platychelus pyropygus* | 79 | 60 | 0 | 0 | 0 | 0 | 139 |
|  | Rutelinid sp.I | 0 | 100 | 3 | 0 | 23 | 0 | 126 |
|  | Rutelinid sp. II | 0 | 2 | 0 | 0 | 0 | 17 | 19 |
|  | Rutelinid sp.III | 179 | 453 | 207 | 2 | 11 | 931 | 1783 |
|  | Rutelinid sp.IV | 0 | 4 | 0 | 0 | 0 | 0 | 4 |
|  | Scarabaeid sp.I | 0 | 0 | 6 | 0 | 0 | 0 | 6 |
|  | Scarabaeid sp.II | 0 | 1 | 0 | 0 | 0 | 0 | 1 |
|  | *Trichostetha capensis* | 0 | 0 | 1 | 0 | 0 | 0 | 1 |
| **Byrrhidae** |  |  |  |  |  |  |  |  |
|  | Byrrhid sp.I | 0 | 0 | 25 | 1 | 1 | 1 | 28 |
|  | Byrrhid sp.II | 0 | 0 | 1 | 0 | 2 | 0 | 3 |
|  | Byrrhid sp.III | 0 | 0 | 6 | 0 | 0 | 4 | 10 |
| **Buprestidae** |  |  |  |  |  |  |  |  |
|  | *Acmaeodera* sp.I | 173 | 0 | 5 | 10 | 11 | 0 | 199 |
|  | *Acmaeodera* sp.II | 16 | 0 | 9 | 0 | 2 | 3 | 30 |
|  | *Acmaeodera* sp.III | 11 | 1 | 1 | 0 | 0 | 2 | 15 |
|  | *Acmaeodera* sp.IV | 0 | 0 | 1 | 0 | 0 | 0 | 1 |
|  | *Acmaeodera* sp.V | 7 | 1 | 26 | 0 | 0 | 1 | 35 |
|  | *Acmaeodera* sp.VI | 3 | 0 | 1 | 0 | 1 | 2 | 7 |
|  | *Acmaeodera* sp.VII | 0 | 0 | 5 | 0 | 0 | 0 | 5 |
|  | *Acmaeodera* sp.VIII | 0 | 0 | 0 | 0 | 1 | 0 | 1 |
|  | *Anthaxia barkeri* | 0 | 0 | 23 | 0 | 0 | 0 | 23 |
|  | *Anthaxia hilaris* | 6 | 1 | 2 | 0 | 0 | 0 | 9 |
|  | *Anthaxia holubi* | 2 | 0 | 0 | 0 | 0 | 1 | 3 |
|  | *Anthaxia tristis* | 15 | 0 | 9 | 0 | 82 | 7 | 113 |
|  | *Nothomorpha* sp. I | 0 | 0 | 0 | 0 | 1 | 2 | 3 |
|  | *Nothomorpha* sp. II | 0 | 0 | 0 | 0 | 2 | 0 | 2 |
|  | *Sphenoptera* sp. | 0 | 0 | 1 | 0 | 0 | 0 | 1 |
| **Elateridae** |  |  |  |  |  |  |  |  |
|  | *Aliteus adspersus* | 0 | 1 | 0 | 0 | 0 | 0 | 1 |
| **Dermestidae** |  |  |  |  |  |  |  |  |
|  | Dermestid sp.I | 17 | 0 | 10 | 0 | 0 | 5 | 32 |
| **Cleridae** |  |  |  |  |  |  |  |  |
|  | Cleriid sp.I | 0 | 0 | 0 | 0 | 0 | 4 | 4 |
| **Melyridae** |  |  |  |  |  |  |  |  |
|  | *Condylops aulicus* | 0 | 1 | 14 | 2 | 2 | 17 | 36 |
|  | *Dasytes costatipennis* | 0 | 2 | 0 | 0 | 0 | 0 | 2 |
|  | *Dasytes plumbeicolor* | 0 | 0 | 90 | 0 | 1 | 24 | 115 |
|  | *Dasytes rubrocupreus* | 0 | 1 | 5 | 3 | 14 | 0 | 23 |
|  | *Melyris limbata* | 0 | 0 | 0 | 0 | 2 | 0 | 2 |
|  | *Melyris nigra* | 0 | 0 | 23 | 0 | 0 | 1 | 24 |
| **Coccinellidae** |  |  |  |  |  |  |  |  |
|  | *Epilachna* sp. | 1 | 0 | 0 | 0 | 0 | 0 | 1 |
|  | *Hippodamia variegata* | 0 | 0 | 0 | 0 | 1 | 1 | 2 |
|  | *Liodalia flavomaculata* | 0 | 0 | 1 | 0 | 0 | 0 | 1 |
| **Meloidae** |  |  |  |  |  |  |  |  |
|  | *Ceroctis capensis* | 1 | 3 | 2 | 0 | 0 | 0 | 6 |
|  | Meloid sp.I | 0 | 3 | 0 | 0 | 0 | 0 | 3 |
|  | *Mylabris (Decatopoma) lunata* | 0 | 0 | 1 | 0 | 0 | 3 | 4 |
|  | *Pagurodactylus angustulus* | 0 | 1 | 0 | 0 | 0 | 1 | 2 |
| **Cerambycidae** |  |  |  |  |  |  |  |  |
|  | *Cerambycid* | 0 | 1 | 1 | 0 | 0 | 0 | 2 |
|  | *Promeces longiceps* | 1 | 2 | 1 | 0 | 0 | 0 | 4 |
| **Chrysomelidae** |  |  |  |  |  |  |  |  |
|  | Chrysomelid sp.I | 9 | 0 | 0 | 0 | 0 | 0 | 9 |
|  | Chrysomelid sp.II | 0 | 0 | 2 | 0 | 1 | 0 | 3 |
|  | Chrysomelid sp.III | 1 | 1 | 2 | 0 | 0 | 0 | 4 |
|  | Chrysomelid sp.IV | 0 | 0 | 4 | 0 | 0 | 0 | 4 |
|  | Chrysomelid sp.V | 1 | 92 | 0 | 0 | 0 | 0 | 93 |
|  | *Creorane erythrocephala* | 0 | 36 | 2 | 0 | 0 | 1 | 39 |
|  | *Eurysthenes* sp. | 2 | 0 | 0 | 0 | 0 | 0 | 2 |
|  | *Exora* cf. *testacea* | 1 | 0 | 0 | 0 | 0 | 3 | 4 |
|  | *Exora* sp. | 81 | 0 | 2 | 0 | 0 | 2 | 85 |
|  | *Pagurodactylus angustissimus* | 8 | 10 | 23 | 1 | 5 | 12 | 59 |
| **Curculionidae** |  |  |  |  |  |  |  |  |
|  | Curculionid sp.I | 0 | 1 | 1 | 0 | 0 | 0 | 2 |
|  | Curculionid sp.II | 2 | 0 | 1 | 0 | 0 | 2 | 5 |
|  | Curculionid sp.III | 0 | 0 | 1 | 0 | 0 | 0 | 1 |
|  |  |  |  |  |  |  |  |  |
| **DIPTERA** |  |  |  |  |  |  |  |  |
|  | Indet. Dipteran | 0 | 0 | 3 | 0 | 1 | 0 | 4 |
| **Bibionidae** |  |  |  |  |  |  |  |  |
|  | *Bibio breviceps* | 15 | 301 | 0 | 0 | 0 | 4 | 320 |
|  | *Bibio* sp. I | 22 | 0 | 591 | 0 | 0 | 1 | 614 |
|  | *Bibio* sp. II | 10 | 193 | 0 | 0 | 0 | 4 | 207 |
|  | *Bibio turneri* | 0 | 0 | 940 | 4 | 4 | 0 | 948 |
|  | *Dilophus* sp.I | 23 | 0 | 511 | 0 | 4 | 0 | 538 |
| **Tabanidae** |  |  |  |  |  |  |  |  |
|  | *Philoliche atricornis* | 28 | 21 | 228 | 3 | 166 | 20 | 466 |
| **Asilidae** |  |  |  |  |  |  |  |  |
|  | Asilid sp.I | 0 | 0 | 0 | 0 | 11 | 0 | 11 |
|  | Asilid sp.II | 0 | 0 | 0 | 0 | 4 | 0 | 4 |
|  | *Neolophonotus sp.* I | 0 | 0 | 7 | 0 | 0 | 1 | 8 |
|  | *Neolophonotus* sp. II | 0 | 1 | 0 | 0 | 0 | 0 | 1 |
|  | *Neolophonotus* sp. III | 0 | 1 | 0 | 0 | 0 | 0 | 1 |
| **Bombyliidae** |  |  |  |  |  |  |  |  |
|  | *Austrolechus* cf. *hirtus* | 0 | 0 | 0 | 1 | 1 | 0 | 2 |
|  | Bombyliid sp.I | 0 | 1 | 0 | 0 | 0 | 0 | 1 |
|  | Bombyliid sp.II | 1 | 17 | 1 | 0 | 0 | 0 | 19 |
|  | Bombyliid sp.III | 0 | 4 | 0 | 0 | 0 | 0 | 4 |
|  | Bombyliid sp.IV | 0 | 10 | 0 | 0 | 0 | 0 | 10 |
|  | *Parisus* sp. | 1 | 0 | 0 | 0 | 0 | 0 | 1 |
|  | *Systoechus* sp.I | 0 | 36 | 0 | 0 | 0 | 0 | 36 |
|  | *Systoechus* sp.II | 0 | 12 | 0 | 0 | 0 | 0 | 12 |
|  | *Systoechus* sp.III | 0 | 15 | 0 | 0 | 0 | 0 | 15 |
| **Empididae** |  |  |  |  |  |  |  |  |
|  | Empidid sp.I | 4 | 0 | 0 | 0 | 0 | 0 | 4 |
|  | Empidid sp.II | 7 | 1 | 0 | 0 | 0 | 0 | 8 |
| **Dolichopodidae** |  |  |  |  |  |  |  |  |
|  | Dolichopodid sp.I | 0 | 0 | 0 | 0 | 0 | 3 | 3 |
| **Syrphidae** |  |  |  |  |  |  |  |  |
|  | *Allograpta fuscotibialis* | 5 | 1 | 0 | 0 | 0 | 0 | 6 |
| **Tephritidae** |  |  |  |  |  |  |  |  |
|  | Tephritid sp.I | 19 | 1 | 3 | 2 | 6 | 2 | 33 |
|  |  |  |  |  |  |  |  |  |
| **Heleomyzidae** |  |  |  |  |  |  |  |  |
|  | Heleomyzid sp.III | 11 | 12 | 0 | 4 | 4 | 2 | 33 |
| **Drosophilidae** |  |  |  |  |  |  |  |  |
|  | Drosophilid sp.I | 3 | 13 | 8 | 7 | 8 | 2 | 41 |
|  | Drosophilid sp.II | 404 | 0 | 0 | 0 | 0 | 0 | 404 |
| **Scathophagidae** |  |  |  |  |  |  |  |  |
|  | *Scathophaga sercoraria soror* | 12 | 18 | 1 | 3 | 8 | 1 | 43 |
|  | *Scathophaga* sp. | 0 | 0 | 1 | 0 | 1 | 0 | 2 |
| **Muscidae** |  |  |  |  |  |  |  |  |
|  | Muscid sp.I | 0 | 0 | 0 | 0 | 1 | 0 | 1 |
|  | Muscid sp.II | 0 | 0 | 0 | 0 | 3 | 0 | 3 |
|  | *Phumosia* sp. | 4 | 20 | 2 | 1 | 34 | 49 | 110 |
| **Calliphoridae** |  |  |  |  |  |  |  |  |
|  | *Chrysoma albiceps* | 0 | 0 | 0 | 0 | 20 | 0 | 20 |
|  | *Chrysomya chloropyga* | 0 | 1 | 13 | 4 | 127 | 3 | 148 |
|  | *Cosmina* sp. | 3 | 0 | 1 | 11 | 19 | 0 | 34 |
|  | *Phumosia* sp. | 1 | 1 | 0 | 0 | 0 | 4 | 6 |
| **Tachinidae** |  |  |  |  |  |  |  |  |
|  | *Gonia* cf. *bimaculata* | 0 | 8 | 0 | 0 | 0 | 0 | 8 |
|  | *Linnaemya* sp. | 0 | 0 | 0 | 0 | 0 | 1 | 1 |
|  | *Zygobothria* sp. | 1 | 0 | 0 | 0 | 0 | 0 | 1 |
|  |  |  |  |  |  |  |  |  |
| **HYMENOPTERA** |  |  |  |  |  |  |  |  |
|  | Parasitoid wasp sp.I | 12 | 215 | 11 | 38 | 41 | 62 | 379 |
|  | Parasitoid wasp sp.II | 0 | 0 | 1 | 0 | 0 | 0 | 1 |
| **Tenthredinidae** |  |  |  |  |  |  |  |  |
|  | Tenthredinid sp.I | 0 | 0 | 1 | 0 | 0 | 0 | 1 |
| **Ichneumonidae** |  |  |  |  |  |  |  |  |
|  | Banchinaeid I | 0 | 0 | 0 | 0 | 1 | 0 | 1 |
|  | Campopleginaeid | 0 | 0 | 0 | 0 | 1 | 0 | 1 |
|  | Cremastinaeid | 0 | 0 | 0 | 0 | 1 | 0 | 1 |
|  | Cryptinaeid sp.I | 0 | 0 | 1 | 0 | 0 | 0 | 1 |
|  | Tersilochinaeid sp.I | 1 | 0 | 0 | 0 | 0 | 0 | 1 |
| **Braconidae** |  |  |  |  |  |  |  |  |
|  | Braconid sp.I | 2 | 0 | 3 | 0 | 0 | 0 | 5 |
|  | Braconid sp.II | 0 | 1 | 0 | 0 | 0 | 0 | 1 |
|  | Braconid sp.III | 0 | 0 | 0 | 0 | 1 | 0 | 1 |
|  | Braconid sp.IV | 0 | 0 | 1 | 0 | 0 | 0 | 1 |
|  | Microgasterinaeid | 0 | 0 | 0 | 0 | 1 | 0 | 1 |
| **Scelionidae** |  |  |  |  |  |  |  |  |
|  | Scelionid sp.I | 0 | 0 | 1 | 0 | 0 | 0 | 1 |
|  | Scelionid sp.II | 3 | 0 | 10 | 0 | 0 | 9 | 22 |
| **Megaspilidae** |  |  |  |  |  |  |  |  |
|  | Megaspilid sp I | 0 | 0 | 0 | 0 | 1 | 6 | 7 |
|  | Megaspilid sp II | 0 | 0 | 1 | 0 | 0 | 0 | 1 |
| **Platygasteridae** |  |  |  |  |  |  |  |  |
|  | Platygasterid sp.I | 0 | 0 | 0 | 2 | 5 | 0 | 7 |
|  | Platygasterid sp.II | 0 | 0 | 0 | 1 | 1 | 0 | 2 |
|  | Platygasterid sp.III | 0 | 0 | 356 | 0 | 2 | 0 | 358 |
| **Figitidae** |  |  |  |  |  |  |  |  |
|  | Eucoilinaeid sp.I | 1 | 0 | 0 | 0 | 0 | 0 | 1 |
|  | Eucoilinaeid sp.II | 0 | 0 | 2 | 0 | 0 | 0 | 2 |
| **Chalcidae** |  |  |  |  |  |  |  |  |
|  | Chalcid sp.I | 0 | 0 | 1 | 0 | 0 | 0 | 1 |
| **Ormyridae** |  |  |  |  |  |  |  |  |
|  | *Ormyrus* sp. | 8 | 0 | 21 | 1 | 31 | 5 | 66 |
| **Pteromalidae** |  |  |  |  |  |  |  |  |
|  | Pteromalid sp.I | 1 | 0 | 0 | 0 | 0 | 0 | 1 |
| **Eulophidae** |  |  |  |  |  |  |  |  |
|  | Eulophid sp.I | 0 | 0 | 2 | 0 | 0 | 0 | 2 |
| **Encyrtidae** |  |  |  |  |  |  |  |  |
|  | Encyrtid sp.I | 1 | 0 | 0 | 0 | 0 | 0 | 1 |
| **Bethylidae** |  |  |  |  |  |  |  |  |
|  | Bethylid sp.I | 0 | 0 | 0 | 1 | 1 | 0 | 2 |
|  | Bethylid sp.II | 1 | 0 | 0 | 0 | 0 | 0 | 1 |
| **Tiphiidae** |  |  |  |  |  |  |  |  |
|  | Tiphiid sp.I | 0 | 0 | 0 | 3 | 3 | 0 | 6 |
|  | Tiphiid sp.II | 0 | 1 | 0 | 0 | 1 | 0 | 2 |
| **Vespidae** |  |  |  |  |  |  |  |  |
|  | Masarinaeid sp.I | 0 | 0 | 0 | 0 | 1 | 0 | 1 |
| **Eumenidae** |  |  |  |  |  |  |  |  |
|  | *Delta hottentotum* | 0 | 0 | 0 | 0 | 0 | 3 | 3 |
| **Sphecidae** |  |  |  |  |  |  |  |  |
|  | *Podalonia* sp. | 0 | 1 | 0 | 0 | 0 | 0 | 1 |
| **Megachilidae** |  |  |  |  |  |  |  |  |
|  | *Immanthium* cf. *junadi* | 0 | 0 | 7 | 0 | 0 | 1 | 8 |
| **Apidae** |  |  |  |  |  |  |  |  |
|  | *Allodape* sp. | 0 | 0 | 1 | 0 | 0 | 0 | 1 |
|  | *Anthophora* cf. *diversipes* | 0 | 1 | 0 | 1 | 1 | 6 | 9 |
|  | *Anthophora* cf. *labrosa* | 0 | 0 | 8 | 3 | 6 | 9 | 26 |
|  | Apid sp.I | 0 | 0 | 0 | 0 | 1 | 0 | 1 |
|  | Apid sp.II | 6 | 0 | 2 | 1 | 1 | 1 | 11 |
|  | *Apis mellifera* | 3 | 12 | 9 | 0 | 1 | 4 | 29 |
|  | *Ctenoceratina* sp. | 0 | 1 | 0 | 0 | 0 | 6 | 7 |
|  | *Sphecodopsis* sp. | 0 | 0 | 7 | 2 | 2 | 0 | 11 |
| **Colletidae** |  |  |  |  |  |  |  |  |
|  | *Scrapter* sp. | 0 | 2 | 0 | 1 | 1 | 0 | 4 |
|  | Colletid sp.I | 0 | 0 | 1 | 0 | 0 | 0 | 1 |
|  | *Polyglossa peringueyi* | 0 | 1 | 0 | 0 | 0 | 0 | 1 |
| **Halictidae** |  |  |  |  |  |  |  |  |
|  | *Ceratina* cf. *subquadrata* | 1 | 0 | 11 | 0 | 0 | 0 | 12 |
|  | *cf Patellapis* sp.I | 1 | 4 | 8 | 1 | 9 | 3 | 26 |
|  | cf. *Patellapis* sp.II | 0 | 0 | 3 | 3 | 18 | 0 | 24 |
|  | Halictid sp.I | 2 | 0 | 1 | 0 | 0 | 1 | 4 |
|  | Halictid sp.II | 0 | 0 | 0 | 0 | 1 | 0 | 1 |
|  | *Halictus andreniformus* | 3 | 0 | 1 | 3 | 3 | 0 | 10 |
|  | *Halictus* cf. *deceptus* | 63 | 18 | 19 | 1 | 5 | 55 | 161 |
|  | *Halictus* cf. *hotoni* | 3 | 0 | 1 | 0 | 2 | 14 | 20 |
|  | *Halictus jucundus* | 103 | 8 | 10 | 0 | 1 | 7 | 129 |
|  | *Halictus vittatus* | 1 | 0 | 0 | 0 | 4 | 0 | 5 |
| **Melittidae** |  |  |  |  |  |  |  |  |
|  | *Redivivoides* sp. | 1 | 0 | 0 | 0 | 0 | 1 | 2 |
|  | *Rediva* sp. | 6 | 4 | 0 | 1 | 1 | 0 | 12 |
| **Formicidae** |  |  |  |  |  |  |  |  |
|  | *Formicid* sp.I | 0 | 0 | 0 | 0 | 1 | 0 | 1 |
|  | *Formicid* sp*.*II | 0 | 0 | 1 | 0 | 0 | 0 | 1 |

### Plant list

Plants from sites in the lowlands of the Cape Floristic region. Further details of the sites can be found in the main text. Aliens, are non-native plants, (indicated by ●). Threat status uses IUCN threatened species categories (CR – critically endangered, EN – Endangered, VU – vulnerable). All information is derived from SANBI online databases (South African National Biodiversity Institute 2007; 2009). Arrangement of families follows that of Germishuizen & Meyer (2003)

|  | **Species** | **Sites** | **Alien** | **Threat Status** |
| --- | --- | --- | --- | --- |
| **PTERIDOPHYTA** |  |  |  |  |
| **Anemiaceae** |  |  |  |  |
|  | *Mohria caffrorum* (L.) Desv. | HE |  |  |
| **GYMNOSPERMS** |  |  |  |  |
| **Pinaceae** |  |  |  |  |
|  | *Pinus pinea* L. | HE | ● |  |
| **DICOTYLEDONS** |  |  |  |  |
| **Aizoaceae** |  |  |  |  |
|  | *Aizoon paniculatum* L. | EL |  |  |
|  | *Aizoon* sp. | VG |  |  |
| **Anacardiaceae** |  |  |  |  |
|  | *Rhus angustifolia* L. | HE |  |  |
|  | *Rhus laevigata sensu* Thunb. | HE |  |  |
|  | *Rhus rosmarinifolia* Vahl | VG |  |  |
|  | *Rhus tomentosa* L. | CO |  |  |
| **Apiaceae** |  |  |  |  |
|  | *Annesorhiza* sp | PH VG |  |  |
|  | *Arctopus echinatus* L. | CO KL PH VG |  |  |
|  | *Centella* sp | EL HE RU |  |  |
|  | *Itasina filifolia* (Thunb.) Raf. | EL |  |  |
|  | *Lichtensteinia* sp. | EL PH |  |  |
|  | *Torilis arvensis* (Huds.) Link | PH VG | ● |  |
| **Apocynaceae** |  |  |  |  |
|  | *Microloma tenuifolium* (L.) K.Schum. | HE |  |  |
| **Asteraceae** |  |  |  |  |
|  | *Arctotheca calendula* (L.) Levyns | EL HE |  |  |
|  | *Arctotheca* sp. | EL |  |  |
|  | *Arctotis incisa* Thunb. | EL |  |  |
|  | *Arctotis* sp. | KL |  |  |
|  | *Athanasia crenata* (L.) L. | HE |  | EN |
|  | *Athanasia crithmifolia* (L.) L. | HE |  |  |
|  | *Athanasia trifurcata* (L.) L. | EL HE KL PH VG |  |  |
|  | *Berkheya armata* (Vahl) Druce | EL KL |  |  |
|  | *Berkheya barbata* (L.f.) Hutch. | VG |  |  |
|  | *Berkheya herbacea* (L.f.) Druce | KL |  |  |
|  | *Carduus* sp. | CO |  |  |
|  | *Chrysanthemoides monilifera* L. (Norl.) | HE |  |  |
|  | *Chrysocoma* sp. | PH |  |  |
|  | *Conyza canadensis* (L.) Cronquist | CO HE VG | ● |  |
|  | *Corymbium* sp. | EL KL RU |  |  |
|  | *Cotula* sp. | PH |  |  |
|  | *Cotula turbinata* L. | EL HE PH VG |  |  |
|  | *Dimorphotheca nudicaulis* (L.) DC. | VG |  |  |
|  | *Dimorphotheca pluvialis* (L.) Moench | EL HE VG |  |  |
|  | *Elytropappus rhinocerotis* (L.f.) Less | CO EL HE KL PH VG |  |  |
|  | *Eriocephalus africanus* L. | EL PH VG |  |  |
|  | *Euryops* sp. | RU |  |  |
|  | *Felicia amoena* (Sch.Bip.) Levyns | EL |  |  |
|  | *Felicia filifolia* (Vent.) Burtt Davy | PH VG |  |  |
|  | *Felicia tenella* (L.) Nees | EL |  |  |
|  | *Gazania serrata* DC. | EL |  |  |
|  | *Gazania* sp. | KL VG |  |  |
|  | *Gerbera* sp. | VG |  |  |
|  | *Gorteria personata* L. | PH |  |  |
|  | *Helichrysum asperum* (Thunb.) Hilliard & B.L.Burtt | EL PH VG |  |  |
|  | *Helichrysum cymosum* (L.) D.Don | CO HE PH VG |  |  |
|  | *Helichrysum dasyanthum* (Willd.) Sweet | CO HE |  |  |
|  | *Helichrysum patulum* (L.) D.Don | CO HE KL PH VG |  |  |
|  | *Helichrysum* sp. | EL HE KL PH VG |  |  |
|  | *Helichrysum teretifolium* (L.) D.Don | KL VG |  |  |
|  | *Hypochaeris radicata* L. | CO EL HE KL VG | ● |  |
|  | *Inuloides tomentosa* (L.f.) B.Nord. |  |  |  |
|  | *Leysera gnaphalodes* (L.) L. | EL |  |  |
|  | *Metalasia* sp.I | CO EL HE KL RU |  |  |
|  | *Metalasia* sp.II | CO |  |  |
|  | *Oedera* sp. L. | RU |  |  |
|  | *Othonna* sp. | EL VG |  |  |
|  | *Picris echioides* L. | CO |  |  |
|  | *Printzia polifolia* (L.) Hutch. | PH |  |  |
|  | *Pseudognaphalium luteo-album* (L.) Hilliard & B.L.Burtt | VG | ● |  |
|  | *Relhania fruticosa* (L.) K.Bremer | EL |  |  |
|  | *Relhania* sp. | PH |  |  |
|  | *Senecio elegans* L. | EL |  |  |
|  | *Senecio hastatus* L. | HE VG |  |  |
|  | *Senecio pterophorus* DC. | CO VG |  |  |
|  | *Senecio puberulus* DC. | CO |  |  |
|  | *Senecio pubigerus* L. | CO HE KL PH VG |  |  |
|  | *Senecio* sp. | PH |  |  |
|  | *Senecio* sp. | EL PH |  |  |
|  | *Senecio thunbergii* Harv. | EL |  |  |
|  | *Sonchus asper* (L.) Hill | CO | ● |  |
|  | *Sonchus oleraceus* L. | CO PH | ● |  |
|  | *Sonchus* sp. | VG | ● |  |
|  | *Stoebe capitata* P.J.Bergius | EL |  |  |
|  | *Stoebe cinerea* Thunb. | CO |  |  |
|  | *Stoebe plumosa* (L.) Thunb. | CO EL HE KL VG |  |  |
|  | *Stoebe* sp. | HE KL RU VG |  |  |
|  | *Tripteris clandestina* Less*.* | EL |  |  |
|  | *Tripteris tomentosa* (L.f.) Less | EL |  |  |
|  | *Ursinia anthemoides* Poir*.* | EL HE KL PH VG |  |  |
|  | *Ursinia discolor* (Less*.*) N.E.Br. | VG |  |  |
|  | *Ursinia* sp. | PH |  |  |
|  | *Ursinia* sp. | HE |  |  |
| **Boraginaceae** |  |  |  |  |
|  | *Echiostachys incanus* (Thunb.) Levyns | EL |  | VU |
|  | *Echium plantagineum* L. | VG |  |  |
|  | *Lobostemon argenteus* (P.J.Bergius) H.Beuk | EL VG |  |  |
|  | *Lobostemon glaber* (Vahl) H.Beuk | EL |  |  |
| **Brassicaceae** |  |  |  |  |
|  | *Heliophila macrosperma* Burch. Ex DC. | EL |  |  |
|  | *Heliophila* sp. L. | PH |  |  |
| **Campanulaceae** |  |  |  |  |
|  | *Merciera* sp. | KL VG |  |  |
|  | *Microcodon* sp. | KL |  |  |
|  | *Wahlenbergia capensis* (L.) A.DC. | EL HE VG |  |  |
| **Caryophyllaceae** |  |  |  |  |
|  | *Cerastium capense* Sond. | EL |  |  |
|  | *Petrorhagia prolifera* (L.) Ball & Heywood | VG |  |  |
|  | *Polycarpon tetraphyllum* (L.) L. | EL | ● |  |
|  | *Silene gallica* L. | CO EL | ● |  |
| **Chenopodiaceae** |  |  |  |  |
|  | *Atriplex semibaccata* R.Br. | PH |  |  |
| **Convolvulaceae** |  |  |  |  |
|  | *Convolvulus capensis* Burm.f. | EL |  |  |
| **Crassulaceae** |  |  |  |  |
|  | *Crassula capensis* (L.) Baill. | KL PH |  |  |
|  | *Crassula ciliata* L. | PH |  |  |
|  | *Crassula fascicularis* Lam. | VG |  |  |
|  | *Crassula glomerata* P.J.Bergius | EL |  |  |
|  | *Crassula* sp. | KL |  |  |
| **Dipsacaceae** |  |  |  |  |
|  | *Scabiosa* sp. | PH |  |  |
| **Droseraceae** |  |  |  |  |
|  | *Drosera cistiflora* L. | EL KL |  |  |
|  | *Drosera pauciflora* Banks*.* Ex DC. | EL KL |  |  |
|  | *Drosera trinervia* Spreng*.* | EL HE |  |  |
| **Ebenaceae** |  |  |  |  |
|  | *Diospyros glabra* (L.) De Winter | KL |  |  |
| **Ericaceae** |  |  |  |  |
|  | *Erica imbricata* L. | HE RU |  |  |
|  | *Erica paniculata* L. | KL |  |  |
|  | *Erica parviflora* L. | HE |  |  |
|  | *Erica plukenetii* L. | HE |  |  |
|  | *Erica* sp.I | HE RU |  |  |
|  | *Erica* sp.II | KL RU |  |  |
|  | *Erica sphaeroidea* Dulfer | HE |  |  |
| **Euphorbiaceae** |  |  |  |  |
|  | *Clutia* sp. | PH VG |  |  |
|  | *Euphorbia erythrina* Link | EL VG |  |  |
|  | *Euphorbia* sp. | CO EL PH VG |  |  |
| **Fabaceae** |  |  |  |  |
|  | *Acacia mearnsii* De Wild | VG | ● |  |
|  | *Acacia saligna* (*Labil*L.) H.L.Wendl. | CO | ● |  |
|  | *Aspalathus aculeata* Thunb. | EL |  |  |
|  | *Aspalathus attenuata* R.Dahlgren | EL |  | EN |
|  | *Aspalathus cephalotes* Thunb. | PH VG |  |  |
|  | *Aspalathus cordata* R.Dahlgren | VG |  |  |
|  | *Aspalathus ericifolia* L. | VG |  |  |
|  | *Aspalathus hispida* Thunb. | HE |  |  |
|  | *Aspalathus* sp.I | EL KL PH VG |  |  |
|  | *Aspalathus* sp.II | HE |  |  |
|  | *Aspalathus spinosa* L. | VG |  |  |
|  | *Aspalathus tridentata* L. | EL |  |  |
|  | *Indigofera* sp.I | PH |  |  |
|  | *Indigofera* sp.II | CO PH VG |  |  |
|  | *Lotononis involucrata s. involucrata* (P.J.Bergius) Benth. | VG |  | VU |
|  | *Lotus* sp. | CO HE |  |  |
|  | *Medicago sativa* L. | EL |  |  |
|  | *Medicago* sp. | HE VG |  |  |
|  | *Otholobium decumbens* (Aiton) C.H.Stirt*.* | HE |  |  |
|  | *Otholobium hirtum* (L.) C.H.Stirt | CO |  |  |
|  | *Podalyria biflora* Lam. | VG |  |  |
|  | *Priestleya* sp. | KL |  |  |
|  | *Psoralea laxa* T.M.Salter | HE |  |  |
|  | *Rafnia* sp. Thunb. | VG |  |  |
|  | *Sutherlandia frutescens* (L.) R.Br. | PH |  |  |
|  | *Trifolium angustifolium* L. | CO | ● |  |
|  | *Vicia* sp. | HE PH | ● |  |
| **Gentianaceae** |  |  |  |  |
|  | *Chironia baccifera* L. | CO HE PH |  |  |
|  | *Sebaea albens* (L.f.) Roem. & Schult. | EL |  |  |
|  | *Sebaea exacoides* (L.) Schinz | KL PH VG |  |  |
|  | *Sebaea* sp. Sol. Ex R.Br. | CO HE PH VG |  |  |
| **Geraniaceae** |  |  |  |  |
|  | *Erodium botrys* (Cav.) Bertol. | EL |  |  |
|  | *Geranium* sp. | CO |  |  |
|  | *Monsonia speciosa* L. | VG |  | EN |
|  | *Pelargonium chamaedryfolium* Jacq. | HE PH |  |  |
|  | *Pelargonium elongatum* (Cav.) Salsib. | CO KL PH VG |  |  |
|  | *Pelargonium myrrhifolium* (L.) L’Hér*.* | CO KL VG |  |  |
|  | *Pelargonium pinnatum* (L.) L’Hér*.* | VG |  |  |
|  | *Pelargonium rapaceum* (L.) L’Hér*.* | VG |  |  |
|  | *Pelargonium* sp. | EL KL |  |  |
|  | *Pelargonium triste* (L.) L’Hér*.* | KL VG |  |  |
| **Lamiaceae** |  |  |  |  |
|  | *Salvia chamelaeagnea* P.J.Bergius | EL |  |  |
| **Lauraceae** |  |  |  |  |
|  | *Cassytha ciliolata* Nees | EL |  |  |
| **Lobeliaceae** |  |  |  |  |
|  | *Cyphia bulbosa* (L.) P.J.Bergius | EL |  |  |
|  | *Cyphia phyteuma* (L.) Willd. | HE KL |  |  |
|  | *Cyphia* sp. | PH |  |  |
|  | *Cyphia volubilis* (Burm.f.) Willd. | EL HE KL PH |  |  |
|  | *Lobelia erinus* L. | HE VG |  |  |
| **Malvaceae** |  |  |  |  |
|  | *Hermannia cuneifolia* Jacq. | PH |  |  |
|  | *Hibiscus aethiopicus* L. | VG |  |  |
| **Mesembryanthemaceae** |  |  |  |  |
|  | *Antimima* sp. | EL |  |  |
|  | *Dorotheanthus bellidiformis* (Burm.f.) N.E.Br. | EL |  |  |
|  | *Erepsia* sp. | EL |  |  |
|  | *Lampranthus elegans* (Jacq.) Schwantes | EL |  |  |
|  | *Lampranthus scaber* (L.) N.E.Br. | EL |  | EN |
|  | *Lampranthus* sp. | VG |  |  |
|  | *Lampranthus spiniformis* (Haw.) N.E.Br. | EL |  |  |
|  | *Phyllobolus* sp. | PH |  |  |
|  | *Ruschia* sp. | PH |  |  |
| **Molluginaceae** |  |  |  |  |
|  | *Adenogramma* sp. | EL |  |  |
|  | *Limeum africanum* L. | EL |  |  |
|  | *Polpoda stipulacea* (F.M.Leight*.*) Adamson | EL |  |  |
| **Montinaceae** |  |  |  |  |
|  | *Montinia caryophyllacea* Thunb. | HE KL |  |  |
| **Orobanchaceae** |  |  |  |  |
|  | *Orobanche ramosa* L. | VG | ● |  |
| **Oxalidaceae** |  |  |  |  |
|  | *Oxalis adspersa* Eckl. & Zeyh. | EL |  |  |
|  | *Oxalis commutata* Sond. | EL KL RU |  |  |
|  | *Oxalis flava* L. | KL PH |  |  |
|  | *Oxalis glabra* Thunb. | EL HE KL |  |  |
|  | *Oxalis hirta* L. | EL |  |  |
|  | *Oxalis lanata* L.f. | HE |  |  |
|  | *Oxalis livida* Jacq. | EL KL PH |  |  |
|  | *Oxalis monophylla* L. | EL |  |  |
|  | *Oxalis obtusa* Jacq. | EL KL PH |  |  |
|  | *Oxalis pes-caprae* L. | EL HE VG |  |  |
|  | *Oxalis polyphylla* Jacq. | EL KL |  |  |
|  | *Oxalis purpurea* L. | EL HE KL PH VG |  |  |
|  | *Oxalis* sp. | EL RU |  |  |
|  | *Oxalis tenuifolia* Jacq. | VG |  |  |
| **Plantaginaceae** |  |  |  |  |
|  | *Plantago lanceolata* L. | CO HE VG |  |  |
| **Polygalaceae** |  |  |  |  |
|  | *Muraltia heisteria* (L.) DC. | EL |  |  |
|  | *Muraltia* sp. | HE |  |  |
|  | *Muraltia trinervia* (L.f.) DC. | EL PH |  |  |
|  | *Polygala bracteolata* L. | VG |  |  |
|  | *Polygala scabra* L. | PH |  |  |
| **Polygonaceae** |  |  |  |  |
|  | *Rumex cordatus* Poir*.* | EL |  |  |
| **Primulaceae** |  |  |  |  |
|  | *Anagallis arvensis* L. | CO HE PH VG |  |  |
| **Proteaceae** |  |  |  |  |
|  | *Leucadendron corymbosum* P.J.Bergius | EL |  | VU |
|  | *Leucadendron lanigerum* H.Beuk*.* ex Meisn. | EL |  | EN |
|  | *Leucadendron salignum* P.J.Bergius | HE KL VG |  |  |
|  | *Leucadendron* sp. | RU |  |  |
|  | *Leucospermum gueinzii* Meisn. | CO |  | EN |
| **Rosaceae** |  |  |  |  |
|  | *Cliffortia polygonifolia* L. | CO HE |  |  |
|  | *Cliffortia ruscifolia* L. | HE |  |  |
|  | *Cliffortia* sp. | RU |  |  |
| **Rubiaceae** |  |  |  |  |
|  | *Anthospermum aethiopicum* L. | VG |  |  |
|  | *Anthospermum galioides* Rchb. | HE |  |  |
|  | *Anthospermum spathulata* Spreng*.* | EL HE PH VG |  |  |
|  | *Galium* sp. | KL PH RU |  |  |
| **Rutaceae** |  |  |  |  |
|  | *Agathosma betulina* (P.J.Bergius) Pillans | HE |  | VU |
|  | *Agathosma* sp. Willd. | PH |  |  |
|  | *Diosma hirsuta* L. | HE VG |  |  |
|  | *Diosma pedicellata* I.Williams | EL |  |  |
|  | *Diosma* sp. | RU |  |  |
| **Santalaceae** |  |  |  |  |
|  | *Thesium* sp. | HE KL VG |  |  |
| **Scrophulariaceae** |  |  |  |  |
|  | *Diascia elongata* Benth. | PH VG |  |  |
|  | *Dischisma arenarium* E.Mey. | EL |  |  |
|  | *Dischisma capitatum* (Thunb.) Choisy | EL |  |  |
|  | *Dischisma* sp. | VG |  |  |
|  | *Nemesia* sp. | PH VG |  |  |
|  | *Polycarena gilioides* Benth. | EL |  |  |
|  | *Selago fruticosa* L. | PH |  |  |
|  | *Selago* sp. | RU |  |  |
|  | *Zaluzianskya divaricata* (Thunb.) *Walp.* | PH VG |  |  |
| **Thymelaeaceae** |  |  |  |  |
|  | *Gnidia laxa* (L.f.) Gilg | HE |  |  |
|  | *Gnidia* sp. | PH |  |  |
|  | *Gnidia* sp. | HE KL VG |  |  |
|  | *Passerina vulgaris* (Meisn.) Thoday | HE |  |  |
|  | *Struthiola dodecandra* (L.) Druce | VG |  |  |
|  | *Struthiola* sp. | RU |  |  |
| **Zygophyllaceae** |  |  |  |  |
|  | *Zygophyllum sessilifolium* L. | EL VG |  |  |
| **MONOCTYLEDONS** |  |  |  |  |
| **Amaryllidaceae** |  |  |  |  |
|  | *Gethyllis* sp. | VG |  |  |
| **Anthericaceae** |  |  |  |  |
|  | *Chlorophytum undulatum* (Jacq.) Oberm*.* | EL |  |  |
| **Asparagaceae** |  |  |  |  |
|  | *Asparagus capensis* L. | PH |  |  |
| **Asphodelaceae** |  |  |  |  |
|  | *Bulbine praemorsa* (Jacq.) Spreng*.* | VG |  |  |
|  | *Bulbinella* sp. Kunth | RU |  |  |
|  | *Trachyandra flexifolia* (L.f.) Kunth | EL |  |  |
|  | *Trachyandra hirsuta* (Thunb.) Kunth | HE |  |  |
|  | *Trachyandra muricata* (L.f.) Kunth | VG |  |  |
|  | *Trachyandra revoluta* (L.) Kunth | EL |  |  |
|  | *Trachyandra* sp. | VG |  |  |
| **Colchicaceae** |  |  |  |  |
|  | *Androcymbium* sp. | PH |  |  |
|  | *Baeometra uniflora* (Jacq.) G.J.Lewis | HE VG |  |  |
|  | *Wurmbea recurva* B.Nord. | EL VG |  |  |
| **Cyperaceae** |  |  |  |  |
|  | *Ficinia indica* (Lam.) Pfeiff. | EL KL VG |  |  |
|  | *Ficinia nigrescens* (Schrad.) J.Raynal | VG |  |  |
|  | *Ficinia* sp.I | HE |  |  |
|  | *Ficinia* sp.II. | CO EL HE KL PH RU |  |  |
|  | *Isolepis* sp. | CO HE |  |  |
|  | *Isolepis verrucosula* (Steud*.*) Nees | EL |  |  |
|  | *Tetraria* sp. | RU |  |  |
|  | *Tetraria ustulata* (L.) C.B.Clarke | HE VG |  |  |
| **Eriospermaceae** |  |  |  |  |
|  | *Eriospermum capense* (L.) Thunb. | VG |  |  |
|  | *Eriospermum* sp. Jacq. Ex Willd | EL VG |  |  |
| **Haemodoraceae** |  |  |  |  |
|  | *Wachendorfia paniculata* Burm. | EL KL |  |  |
|  | *Wachendorfia* sp. | EL |  |  |
| **Hyacinthacaea** |  |  |  |  |
|  | *Albuca juncifolia* (Hilliard & B.L.Burtt) | EL |  |  |
|  | *Albuca* sp. | EL VG |  |  |
|  | *Drimia capensis* (Burm.f.) Wijnlands | PH |  |  |
|  | *Drimia physodes* (Jacq.) Jessop | EL |  |  |
|  | *Lachenalia contaminata* Aiton | EL |  |  |
|  | *Lachenalia pallida* Aiton | EL |  |  |
|  | *Lachenalia polyphylla* Baker | EL |  |  |
|  | *Lachenalia* sp. | EL VG |  |  |
|  | *Lachenalia unifolia* Jacq. | EL |  |  |
|  | *Ornithogalum hispidum* Hornem | KL RU |  |  |
|  | *Ornithogalum* sp. | PH |  |  |
|  | *Ornithogalum thyrsoides* Jacq. | EL PH VG |  |  |
| **Hypoxidacaea** |  |  |  |  |
|  | *Empodium plicatum* (Thunb.) Garside | EL |  |  |
|  | *Empodium* sp. | VG |  |  |
|  | *Spiloxene capensis* (L.) Garside | EL |  |  |
|  | *Spiloxene* sp. | HE PH |  |  |
| **Iridaceae** |  |  |  |  |
|  | *Babiana* sp. | EL KL VG |  |  |
|  | *Bobartia* sp. | HE RU VG |  |  |
|  | *Ferraria* sp. Burm. ex Mill. | EL |  |  |
|  | *Geissorhiza aspera* Goldblatt | EL HE KL PH |  |  |
|  | *Geissorhiza imbricata* (D.Delaroche) Ker Gawl. | EL |  |  |
|  | *Geissorhiza* sp. | EL PH RU |  |  |
|  | *Gladiolus alatus* L. | EL |  |  |
|  | *Gladiolus carneus* D.Delaroche | VG |  |  |
|  | *Gladiolus* sp. | KL RU |  |  |
|  | *Hesperantha falcata* (L.f.) Ker Gawl. | HE VG |  |  |
|  | *Hesperantha pilosa* (L.f.) Ker Gawl. | EL |  |  |
|  | *Hesperantha radiata* (Jacq.) Ker Gawl. | PH |  |  |
|  | *Hesperantha* sp. | RU |  |  |
|  | *Ixia lutea* Eckl. | EL |  |  |
|  | *Ixia* sp. | PH VG |  |  |
|  | *Lapeirousia azurea* (Eckl. ex Baker) Goldblatt | EL |  |  |
|  | *Lapeirousia* sp. | VG |  |  |
|  | *Micranthus alopecuroides* (L.) Rothm. | HE VG |  |  |
|  | *Micranthus junceus* (Baker) N.E.Br. | KL |  |  |
|  | *Micranthus tubulosus* (Burm.) N.E.Br. | EL KL VG |  |  |
|  | *Moraea angusta* (Thunb.) Ker Gawl. | VG |  |  |
|  | *Moraea bituminosa* (L.f.) Ker Gawl. | VG |  |  |
|  | *Moraea galaxia* (L.f.) Goldblatt & J.C.Manning | EL KL PH VG |  |  |
|  | *Moraea inconspicua* Goldblatt | PH VG |  |  |
|  | *Moraea lewisiae* (Goldblatt) Goldblatt | EL |  |  |
|  | *Moraea* sp. | EL HE KL PH RU VG |  |  |
|  | *Moraea tricuspidata* (L.f.) G.J.Lewis | HE |  |  |
|  | *Moraea tripetala* (L.f.) Ker Gawl. | RU |  |  |
|  | *Moraea umbellata umbellata* | EL |  |  |
|  | *Moraea villosa s. villosa* (Ker Gawl.) Ker Gawl. | EL |  |  |
|  | *Romulea* sp. | EL RU |  |  |
|  | *Sparaxis* sp. | KL PH |  |  |
|  | *Tritonia crispa* (L.f.) Ker Gawl. | VG |  |  |
|  | *Tritonia* sp. | PH VG |  |  |
|  | *Watsonia* sp. | HE VG |  |  |
| **Juncaceae** |  |  |  |  |
|  | *Juncus cephalotes* Thunb. | EL |  |  |
| **Orchidaceae** |  |  |  |  |
|  | *Corycium orobanchoides* (L.f.) Sw. | VG |  |  |
|  | *Disa bracteata* Sw. | HE |  |  |
|  | *Disperis* sp. | PH |  |  |
|  | *Disperis villosa* (L.f.) | EL |  |  |
|  | *Holothrix* sp. *Rich.* Ex Lindl. | PH VG |  |  |
|  | *Holothrix villosa* Lindl. | RU |  |  |
|  | *Pterygodium alatum* (Thunb.) Sw. | EL KL |  |  |
|  | *Pterygodium catholicum* (L.) Sw. | HE KL PH |  |  |
|  | *Satyrium* sp. | RU |  |  |
| **Poaceae** |  |  |  |  |
|  | *Aira cupaniana* Guss*.* | KL PH VG |  |  |
|  | *Avena barbata* Pott ex Link | HE |  |  |
|  | *Avena* sp. | CO |  |  |
|  | *Brachypodium distachyon* (L.) P.Beauv. | HE PH |  |  |
|  | *Briza maxima* L. | CO EL HE PH VG | ● |  |
|  | *Briza minor* L. | EL HE VG | ● |  |
|  | *Bromus molliformus* | CO HE | ● |  |
|  | *Bromus pectinatus* Thunb. | HE PH |  |  |
|  | *Bromus rigidus* Roth. | HE | ● |  |
|  | *Bromus* sp. | HE |  |  |
|  | *Cymbopogon marginatus* (Steud*.*) Stapf ex Burtt Davy | EL HE VG |  |  |
|  | *Cynodon dactylon* (L.) Pers | EL |  |  |
|  | *Ehrharta calycina* Sm. | EL HE PH VG |  |  |
|  | *Ehrharta capensis* Thunb. | EL KL PH VG |  |  |
|  | *Ehrharta* sp. | PH |  |  |
|  | *Eragrostis curvula* (Schrad.) Nees | EL VG |  |  |
|  | *Eragrostis* sp. | VG |  |  |
|  | *Heteropogon contortus* Pers*.* | VG |  |  |
|  | *Lolium perenne* L. | CO EL | ● |  |
|  | *Merxmuellera* sp. | KL |  |  |
|  | *Merxmuellera stricta* (Schrad.) Conert | HE PH VG |  |  |
|  | *Pennisetum clandestinum* Hochst. ex Chiov | CO | ● |  |
|  | *Pentaschistis airoides s. airoides* (Nees) Stapf | HE VG |  |  |
|  | *Pentaschistis* sp.I | KL |  |  |
|  | *Pentaschistis* sp.I | KL PH RU VG |  |  |
|  | *Setaria* sp. | VG |  |  |
|  | *Stipagrostis zeyheri sub*sp. *zeyheri* (Nees) De Winter | EL |  |  |
|  | *Themeda triandra* Forssk. | EL VG |  |  |
|  | *Tribolium hispidum* (Thunb.) Desv. | EL VG |  |  |
|  | *Tribolium* sp. Desv. | PH |  |  |
|  | *Tribolium uniolae* (L.f.) Renvoize | EL HE KL VG |  |  |
|  | *Vulpia myuros* (L.) C.C.Gmel. | CO EL HE PH VG | ● |  |
| **Restionaceae** |  |  |  |  |
|  | *Elegia* sp. | EL RU |  |  |
|  | *Hypodiscus* sp. | RU |  |  |
|  | *Ischyrolepis capensis* (L.) H.P.Linder | EL KL VG |  |  |
|  | *Ischyrolepis* sp. | EL KL RU |  |  |
|  | *Ischyrolepis* sp. | RU |  |  |
|  | Restio sp. | KL RU VG |  |  |
|  | *Thamnochortus* sp. | EL KL RU |  |  |
|  | *Willdenowia* sp. | RU |  |  |
| **Tecophilaeaceae** |  |  |  |  |
|  | *Cyanella hyacinthoides* L. | EL HE PH VG |  |  |
|  | *Cyanella lutea* L.f. | KL PH |  |  |

### References

Germishuizen, G. & Meyer, N. L. (2003) *Plants of Southern Africa: an annotated checklist*. National Botanical Institute, Pretoria.

Mucina, L. & Rutherford, M. (2006) *The vegetation of South Africa, Lesotho and Swaziland*. South African National Biodiversity Institute, Pretoria.

Scholtz, C.H. & Holm, E. (2008) *Insects of Southern Africa*. Protea Book House, Pretoria.

South African National Biodiversity Institute (2007) *Plants of Southern Africa (POSA): and online checklist. Version 2.0*. <http://posa.sanbi.org/>. Accessed: 19-4-2009.

South African National Biodiversity Institute (2009) *SIBIS - SANBI's integrated biodiversity information system*. <http://sibis.sanbi.org>. Accessed: 3-9-2009.
