# Supplementary material for: Species turnover in plants does not predict turnover in flower-visiting insects
Source: PeerJ. 2018 Dec 21;6:e6139. doi: 10.7717/peerj.6139 (PMC6305123; doi:10.7717/peerj.6139)
Supplement: Supplemental Information 3 — Description of the results of the pan and plot similarities. [file peerj-06-6139-s003.docx]

*Pan and plot assemblage similarity*

The sixteen plots separated according to their plant (Figure S1a) and insect assemblages (Figure S1b). For plants, eight significantly different branches could be discerned. Although groupings generally followed the spatial separation of the plots, with nearby plots from the same sites grouping together, there were exceptions, for example plot KL1 which grouped with sites from EL1-4, almost 70 km away, and plot EL2 which was distinct from all other plots (see Appendix S1 in Supporting Information for distances between plots). There was a greater degree of similarity between anthophile assemblages at plots with the first major division occurring at 19.23% dissimilarity, and the grouping of plots was much more spatially distinct. Indeed, there were seven significant branches, with no intermingling of distant plots (Figure S1b).
